# The adenosinergic signaling in the pathogenesis and treatment of multiple sclerosis

**DOI:** 10.3389/fimmu.2022.946698

**Published:** 2022-07-28

**Authors:** Eduardo Duarte-Silva, Henning Ulrich, Ágatha Oliveira-Giacomelli, Hans-Peter Hartung, Sven G. Meuth, Christina Alves Peixoto

**Affiliations:** ^1^ Laboratory of Ultrastructure, Aggeu Magalhães Institute (IAM), Recife, Brazil; ^2^ Postgraduate Program in Biosciences and Biotechnology for Health (PPGBBS), Oswaldo Cruz Foundation (FIOCRUZ-PE)/Aggeu Magalhães Institute (IAM), Recife, Brazil; ^3^ Network of Immunity in Infection, Malignancy and Autoimmunity (NIIMA), Universal Scientific Education and Research Network (USERN), Recife, Brazil; ^4^ Department of Neurology, Medical Faculty, University Hospital Düsseldorf, Düsseldorf, Germany; ^5^ Department of Biochemistry, Institute of Chemistry, University of São Paulo, São Paulo, Brazil; ^6^ Brain and Mind Center, University of Sydney, Sydney, NSW, Australia; ^7^ Department of Neurology, Palacky University Olomouc, Olomouc, Czechia; ^8^ Department of Neurology, Medical University of Vienna, Vienna, Austria; ^9^ National Institute of Science and Technology on Neuroimmunomodulation (INCT-NIM), Oswaldo Cruz Institute, Oswaldo Cruz Foundation, Rio de Janeiro, Brazil

**Keywords:** experimental autoimmune encephalomyelitis (EAE), multiple sclerosis (MS), adenosinergic signaling, adenosine, adenosine receptors, therapies

## Abstract

Multiple sclerosis (MS) is a highly disabling, progressive neurodegenerative disease with no curative treatment available. Although significant progress has been made in understanding how MS develops, there remain aspects of disease pathogenesis that are yet to be fully elucidated. In this regard, studies have shown that dysfunctional adenosinergic signaling plays a pivotal role, as patients with MS have altered levels adenosine (ADO), adenosine receptors and proteins involved in the generation and termination of ADO signaling, such as CD39 and adenosine deaminase (ADA). We have therefore performed a literature review regarding the involvement of the adenosinergic system in the development of MS and propose mechanisms by which the modulation of this system can support drug development and repurposing.

## Introduction

Multiple Sclerosis (MS) is a chronic neurodegenerative disease of the central nervous system (CNS) characterized by demyelination, neuroaxonal degeneration and autoimmunity (1). Disease pathogenesis involves the complex interplay of numerous signaling pathways, including for example those driving apoptosis, inflammation and excitotoxicity, with the components of these pathways offering interesting targets for drug discovery or repurposing (2).

The adenosinergic signaling pathway is comprised of molecules and receptors involved in the conversion of adenosine triphosphate (ATP) and adenosine diphosphate (ADP) into adenosine (ADO), and includes the subsequent reuptake and metabolism of ADO intracellularly (3). After being released into the extracellular space, ATP and ADP are converted to adenosine monophosphate (AMP) by the ectoenzyme CD39. AMP is then converted into ADO by the enzyme CD73. Alternatively, controlled release of ADO by the cell may occur. Once extracellular, ADO can now bind to its four cognate purinergic receptors, the P1 receptors (P1Rs), which are G protein-coupled receptors (GPCRs): adenosine A_1_ receptor (A1R), adenosine A_2A_ receptor (A2AR), adenosine A_2B_ receptor (A2BR) and adenosine A_3_ receptor (A3R). Following receptor binding, ADO triggers complex signaling cascades. For instance, signaling through G_s_ (A2AR and A2BR) leads to the activation of adenylate cyclase (AC), production of cyclic adenosine monophosphate (cAMP) and the subsequent activation of protein kinase A (PKA), which acts on downstream targets to modulate gene expression. On the other hand, signaling through G_i/o_ (A1R and A3R) results in the inhibition of AC and cAMP production. Of note, activation of mitogen-activated protein kinase (MAPK) is a downstream step common to all adenosine receptors (3).

After binding to its receptors and triggering its intracellular effects, ADO is captured by equilibrative nucleoside transporters (ENTs) 1 and 2 and transported into the cell, where it is either converted to inosine by the enzyme adenosine deaminase (ADA) or to AMP by adenosine kinase (4). The nature of adenosine or ADO release is critical in determining whether P1R signaling is beneficial or detrimental. Specifically, an acute increase in the adenosine tone or increase in the extracellular levels of ADO is protective as it suppresses the immune response as well as the detrimental effects of an overzealous immune response, and thus helps in the reestablishment of tissue homeostasis and healing. However, a chronically elevated extracellular ADO level is associated with detrimental effects and pathological conditions, such as inflammatory tissue trauma, inflammation, hypoxia, and cancer (3).

Notably, purinergic receptors are widely involved in inflammatory processes (4, 5) and the literature demonstrates that P1 receptors can target neuromodulation and inhibition of inflammation, including in MS models (4, 6, 7). Thus, targeting this pathway may be a promising therapeutic strategy. In addition, it has been demonstrated that MS patients have decreased levels of plasma ADO (8). Therefore, we aim to present an updated literature review regarding the adenosinergic signaling pathway in the pathogenesis and treatment of MS. Harnessing the knowledge of this pathway is a promising strategy for the discovery of new molecular targets and the development of new drugs.

## Adenosine A_1_ receptors

A1R is abundantly expressed in the CNS, exerting inhibitory effects on glutamatergic and GABAergic synapses in particular (6). It participates in the finely-tuned regulation of excitatory neurotransmission, preventing its excess (6). In addition to its expression in neurons, glia and brain endothelial cells (9), A1R is also expressed in immune cells, aiding the modulation of inflammation (3). Interestingly, A1R expression is hindered by PICs, such as TNF-α and IL-1β (10) and is found to be decreased at both the RNA and protein level in MS patients, either in peripheral blood mononuclear cells (PBMCs) – specifically in monocytes, or in the microglia/macrophages within brain tissue (11). On the one hand, A1R activation in monocytoid cells triggered inhibition of TNF-α expression (11) and activation of A1R by its agonist R-phenylisopropyladenosine (R-PIA) was shown to block the secretion of IL-6 from PBMCs from MS patients (8). This suggests A1R is an important immunomodulator and helps to partially explain why increased levels of PICs are observed in MS. However, A1R knockout mice in which EAE was induced demonstrated a more severe form of disease compared to wild type (12). The knockout mice had increased microglial activation, PIC gene expression and demyelination, suggesting a protective role of A1R activity (12). Furthermore, low levels of A1R were found in the peripheral lymphocytes of MS patients, which highlights the importance of the infiltration of monocytes/macrophages and microglia over the infiltration of encephalitogenic lymphocytes in the pathogenesis and progression of MS (11). Of note, extensive infiltration of immune cells into the brain, a core feature of relapsing-remitting multiple sclerosis (RRMS), is due to increased permeability of the blood-brain barrier (BBB) (2, 13).

Interestingly, adenosinergic signaling is involved in the modulation of BBB permeability (9). Activation of all four adenosine receptors (ARs) by a non-specific AR agonist increased BBB permeability to labeled dextran for up to 18 hours (h) *in vivo*. Similar results were obtained when specific A1R and A2AR agonists were used, especially in combination, which resulted in even greater BBB permeability (9). Although these results are of relevance to AD (9) and other conditions in which the BBB impedes access of therapeutics to the CNS – including the progressive form of MS, in RRMS it may be said that using A1R and A2AR antagonists is a better therapeutic strategy to reduce BBB permeability, which is in fact observed after treatment with immunomodulatory drugs in MS (14). Interestingly, dexamethasone (DEX), a drug often used to treat MS relapses, exerts anti-inflammatory effects by modulating the expression of A1R. More specifically, it was shown that treatment of monocytoid cells with DEX caused an increase in the expression of A1R mRNA (10). Moreover, the mechanism of A1R levels being decreased in MS and thus contributing to prolonged neuroinflammation was also partially determined: MS patients have augmented levels of β-arrestin, especially brain macrophages/microglia, and overexpression of this molecule in monocytoid cells triggered a decrease in A1R levels. In mice, β-arrestin levels are also increased at the onset and during the early disease course (at day post-induction 3). Not surprisingly, DEX also attenuates inflammation by reducing the levels of β-arrestin in EAE mice (10). Considering that β-arrestin physically interacts with A1R receptors and that β-arrestin levels can be upregulated by inflammation (10), A1R desensitization mediated by β-arrestin may underpin the dysregulated adenosinergic signaling and neuroinflammation observed in MS. This dysregulation occurs early in MS pathogenesis, in the inflammatory phase of disease (15), raising the possibility that A1R agonists, when used early, may inhibit the excessive inflammatory response and prevent or delay the development of the neurodegenerative phase. Paradoxically, these anti-inflammatory effects would take place at the expense of an increased BBB permeability that, depending on the degree of permeability, may also contribute to a certain extent to an increased inflammatory response in the CNS. Further *in vitro* data are therefore needed to establish the best strategy, balancing the benefits against the risks.

Demyelination is a core feature of MS and therapies aiming to promote myelination or prevent demyelination are therefore of key importance in tackling the disease. In this vein, the adenosinergic system is also implicated in the modulation of oligodendrocytes [extensively reviewed in (16, 17)]. Briefly, it has been demonstrated that ADO signaling *via* A1R in oligodendrocyte progenitor cells (OPCs) stimulated their maturation and migration and promoted myelination (17). Furthermore, an A1R agonist was shown to prevent extension of demyelination induced by lysolecithin (18). These results further suggest that agonism of A1R is likely to be a promising therapeutic strategy in MS.

An interesting question that remains be to answered by future studies is whether modulation of A1R on pre-synaptic and post-synaptic neurons could be useful in the regulation of glutamate (Glu) neurotransmission and subsequently induced Glu excitotoxicity, which is a key feature of MS pathology (19). A1R signaling inhibits the release of Glu and limits the influx of Ca^2+^ by the pre-synaptic neuron, as well as decreases the activity of NMDAR in the post-synaptic neuron, thus limiting Glu neurotransmission (6). However, as Glu is also implicated in synaptic plasticity and long-term potentiation (LTP) and long-term depression (LTD), drugs targeting this system should be used with caution and specificity.

Inflammation in MS can also occur due to K^+^ channel dysfunction. For instance, MS patients were shown to have increased expression of the K^+^ channel K_2P_5.1 (or TASK2) in CD4^+^ and CD8^+^ T cells (20) and K_2P_18.1. was shown to regulate Regulatory T cells (Tregs) development (21). Moreover, there is also a role for voltage-gated K^+^ channels in neuroinflammation, as the pharmacological inhibition of Kv1.1 attenuated EAE development (22). Interestingly, A1R triggers activation of K2P (23), but the implication of this crosstalk in the context of neuroinflammation, neurodegeneration and autoimmunity remains elusive.

In conclusion, the modulation of A1R activity for MS treatment has conflicting effects depending, for instance, on cell type, although the evidence suggests the effect is mostly beneficial. While A1R activation seems to decrease PIC release in microglia and macrophages, which could decrease the inflammatory response and promote remyelination, it also increases BBB permeability and consequent peripheral immune cell infiltration. This conflicting yin-yang nature of A1R pharmacology in MS makes it difficult to ensure which therapeutic approach, whether using agonists or antagonists, is better for MS treatment. However, these aspects are yet to be characterized by future pre-clinical and clinical studies. It is likely further study in this area will aid in the treatment of MS and deepen our understanding of its pathogenesis.

## Adenosine A_2_ receptors

Similar to A1R, A2R is also widely expressed in the CNS, specifically in synapses, where it also participates in the regulation of excitatory neurotransmission. However, A2R in contrast to A1R modulates synaptic plasticity by facilitating excitatory transmission, which occurs through the inhibition of the pre-synaptic inhibitors A1R and cannabinoid type 1 receptor (CB1R) (6). More importantly, it promotes LTP. However, excessive A2R signaling has been linked to aging, memory impairment and synaptotoxicity (6). In addition to its distribution in the CNS, A2R are also abundantly expressed in immune cells (5).

### Adenosine A_2a_ Receptors

A2AR plays an important role in the pathophysiology of MS. Similar to A1R knockout mice, A2AR knockout mice presented a more severe course of EAE compared to wild type (24), which is likely to be caused by an augmented number of cells secreting IFN-γ, IL-17 and granulocyte-macrophage colony-stimulating factor (GM-CSF) (25). More specifically, increased axonal damage, extensive demyelination, increased number of microglia and astrocytes and splenic CD4^+^ T cells were detected in mice lacking A2AR (26). Furthermore, upregulation of PICs and downregulation of anti-inflammatory cytokines, such as TGF-β and IL-10 were also detected in the brain and spinal cord (26), which is consistent with the finding that A2AR exerts anti-inflammatory effects (27). However, A2AR was found to be highly expressed on the endothelium of MS patients in areas of white matter injury (28). Lymphocytes from MS patients were shown to possess increased gene and protein expression levels of A2AR (27), which is also observed in the chronic phase of the MS murine model, EAE (25). Moreover, treatment of these lymphocytes with a specific A2AR agonist led to inhibition of the secretion of PICs, such as IL-6, IFN-γ and IL-17, and inhibition of VLA-4 protein expression levels and NF-κB activation (27).

In addition to the anti-inflammatory effects, treatment of EAE mice with an A2AR agonist also caused inhibition of the ability of microglia and macrophages to phagocytose and migrate *in vitro* and inhibited their activation *in vivo* (29, 30). Impaired migration capacity was also reported to occur in CD4^+^ T lymphocytes treated with this agonist (25). However, similar findings were also reported after the administration of an A2AR antagonist. It was shown that A2AR antagonism after disease onset reduced immune cell infiltration, demyelination and IFN-γ levels in the CNS while administration in the asymptomatic phase of disease did not reduce disease severity. This suggests that there is a therapeutic window that needs to be considered when using adenosinergic signaling modulators to tackle MS. This is also corroborated by findings showing that the activation of A2AR early in the disease course reduced its severity, but it worsened when activation takes place late in the course of disease (25).

Interestingly, contrary to what has been observed with A1R, A2AR stimulation is associated with an inhibition of oligodendrocyte progenitor cell (OPC) differentiation (17). Similarly to A1R, A2AR has also been implicated as a promoter of BBB permeability (9), although opposite findings were reported. A2AR agonism prevented the reduction in the levels of zonula occludens-1 (ZO-1) and claudin-5, tight junction proteins that are crucial for BBB permeability maintenance, induced by Th1 cytokines *in vitro.* Similarly, it also caused a decrease in BBB permeability *in vivo*, as observed by reduced uptake of fluorescent tracer into the CNS (28). Further, and as expected, A2AR plays an important role in the infiltration of immune cells into the CNS. It was shown that A2AR agonism led to increased expression of the chemokine CX3CL1/fractalkine, which is also upregulated in EAE mice on day post induction (dpi) 10. An inverse effect was observed after the administration of an A2AR antagonist. Interestingly, A2AR activation led to CX3CL1 expression on choroid plexus cells, allowing the massive infiltration of CD4^+^ and CD8^+^ T cells and macrophages into the CNS (31). More recent data also showed that pharmacological inhibition and genetic perturbation of A2AR reduced immune cell infiltration *via* the choroid plexus (CP) with consequent attenuation of EAE progression. However, agonism resulted in increased permeability in the CP and high expression of CCL20 gene (32). Interestingly, A2AR antagonism downregulated CCL20 expression in CP and reduced Th17 infiltration in the cerebrospinal fluid (CSF) and spinal cord of EAE mice (32).

These findings demonstrate that A2AR receptor activation induces conflicting effects, possibly depending on the time point of MS development. In early stages, A2AR receptor activation induces protective effects and, in later stages, induces detrimental effects. Moreover, although the literature reports anti-inflammatory effects of A2AR activation, its expression was found to be increased in areas of injured brain tissue and the lymphocytes of MS patients. Thus, although these changes in A2AR levels were regarded as a compensatory mechanism, whether they correlate with an improvement in MS symptoms or just act to prevent the exacerbation of symptoms is yet to be determined. Furthermore, A2AR activation may lead to immune cell infiltration into the CNS *via* upregulation of CX3CL1 on endothelium/choroid plexus, which may facilitate the establishment of CNS inflammation and thereby worsen disease.

### Adenosine A_2b_ Receptors

A2BR is extensively expressed on immune cells, such as mast cells, and is associated with pro-inflammatory actions. ADO binding to A2BR occurs only at high concentrations (10 µM), which is observed during pathological conditions, such as inflammation, hypoxia and trauma (5). Of note, ADO at physiological levels (0.01-1 µM) can bind to A1R, A2AR and A3R (5).

PBMCs of MS patients have increased expression of A2BR (33). In mice, high levels of A2BR in the spleen and in lymph nodes has also been documented (33). Interestingly, administration of an A2BR antagonist alleviates disease severity, demyelination and the infiltration of CD4^+^ T cells, Th1 and Th17 cells in an EAE model. Moreover, a decrease in Th1 and Th17 responses as well as reduced levels of IFN-γ, IL-6, IL17A, IL-17F, IL-22 and IL-23 were detected in the spleen. In addition to the effects of A2BR inhibition on lymphocytes, reduced production of IL-6 by dendritic cells has also been reported (33). A2BR therefore appears to be an important target in MS since its activation is associated with pathological conditions, in which ADO production exceeds ADO reuptake and the termination of ADO signaling. Moreover, targeting this receptor with specific antagonists is promising as it modulates the development of the Th17 response and can thus alleviate the autoimmune response. Compared to A1R and A2AR, the literature to date does not provide conflicting or paradoxical findings, making this receptor an attractive target in MS. However, due to the paucity of data regarding its effects on other aspects of MS pathology, such as the BBB permeability, caution should be taken when using A2BR antagonists.

## Adenosine A_3_ receptors

A3R is distributed in immune cells and has a prominent role in the regulation of immunity (5). Although the role of ADORA3 is better characterized in cancer research (3), its role in the pathogenesis of MS is undefined and thus data on this receptor are limited. It is known that A3R levels are increased in the spinal cord of EAE mice (33). However, ADORA3 receptor activation reduced mouse microglial TNF-α release in an *in vitro* model of neuroinflammation triggered by lipopolysaccharide (34). More studies are needed to allow precise characterization of the functional effects of this receptor in the development of MS, which may then contribute to the design of novel therapies.

## Adenosine receptor antagonists

A1R and A2AR, although they exert different effects, are often evaluated together as caffeine is a non-selective A1R/A2AR antagonist. Administration of caffeine has a beneficial effect in experimental autoimmune encephalomyelitis (EAE), one of the most commonly used animal models of MS. It was demonstrated that chronic caffeine administration led to reduced infiltration of immune cells into the cerebral cortex and spinal cord and decreased demyelination in EAE mice. Furthermore, caffeine ameliorated EAE by reducing the expression of pro-inflammatory cytokines (PICs), such as IFN-γ, and increasing the expression of A1R in the spinal cord (35). More specifically, the beneficial effects of caffeine were mostly observed in the effector phase of disease, where neurodegeneration is more prominent (24). Notably, reduced infiltration of immune cells and demyelination and increased levels of A1R were observed after chronic caffeine administration. However, when caffeine was administered 10 days prior to EAE induction, it did not have any effect on the disease course (24), which raises the question whether the consumption of caffeine is effective in preventing EAE from occurring.

It has been reported that moderate and regular caffeine consumption is associated with an overall lower susceptibility for developing AD, cognitive decline and dementia (36). In fact, human studies explored whether regular consumption of caffeine is associated with a lower risk of developing MS. A Swedish study revealed a reduced risk for MS development in those who consumed high amounts of coffee and a Belgian study demonstrated that daily coffee intake delayed MS symptom onset (37, 38). However, studies conducted in the United States, Australia and Serbia did not confirm this relationship, the latter in fact reporting that patients with MS consume comparatively more coffee (39–41). In addition to these studies, the beneficial effects of caffeine in MS patients with fatigue, a particularly debilitating symptom of MS, were evaluated. It was demonstrated that caffeine consumption increased quality of life by increasing patients’ ability to perform activities of daily living (42).

## CD39, CD73, regulatory T cells and MS regulation

Tregs have been widely acknowledged for their role in suppressing an overactive immune response. Interestingly, this can be achieved by the expression of the ectonucleotidase CD39 on these cells, allowing them to suppress, for instance, the production of IL-17 (43), an important cytokine in the pathogenesis of MS. Moreover, ADO produced by the coordinated action of CD39 and CD73 on Tregs inhibited Th1 and Th2 responses (44, 45). However, numbers and functionality of CD39^+^ Tregs cells were reported to be decreased and compromised in a subset of RRMS patients, making these cells incapable of suppressing IL-17 production *in vitro* (43). Intriguingly, increased activity of CD39 as well as increased CD39 mRNA levels (46) were detected in PBMCs and Tregs in another cohort of RRMS patients in comparison to healthy subjects (46, 47). Notably, at the time of the study these patients were experiencing an “acute exacerbation” (47) or were “relapsing” (46), which may explain these contradictory findings. Increased levels of CD39 in PBMCs of RRMS patients in remission was also reported (48). Of note, expansion of CD39^+^Tregs is induced after treatment with immunomodulatory drugs, such as fingolimod (49) or methylprednisolone (46), or alemtuzumab (50), in RRMS patients or with teriflunomide in EAE mice (51), which suggests that these cells may inhibit the inflammatory response and contribute to the remission phase of the disease *via* the production of ADO. Notably, a positive correlation was found between CD39^+^ Tregs and Th17 lymphocytes in RRMS patients in remission (52). Interestingly, this expansion of CD39+ Tregs is also observed after the exposure of T lymphocytes from RRMS patients to serotonin (5-HT) (53). Surprisingly, CD39 expression on CD4^+^ T cells and increased proportions of this cell population can also be induced by polysaccharide A (PSA), a molecule derived from *Bacteroides fragilis*, a commensal microorganism that inhabits the gut (54–56), which highlights the important role of the gut microbiota in the pathophysiology of MS (57, 58), especially in the context of ADO signaling.

In monophasic EAE, increased activity of CD39 and CD73 were observed in the rat spinal cord, at dpi 15 and 25, correlating with extensive immune cell infiltration (59). Similar findings were also reported in SJL/J mice at the peak of disease (25) and in Dark Agouti rats at disease onset and disease peak (60). Furthermore, increased levels of CD39 and CD73 were detected in the CSF of RRMS patients, although TNF-α levels were also increased. However, an inverse pattern was reported to occur in the PBMC of the same patients (61). Interestingly, the levels of the adenosine deaminase (ADA) (48) were shown to be decreased in lymphocytes of RRMS patients, which suggests ADO signaling termination may be compromised and that there may be an intracellular accumulation of ADO in these cells, since this enzyme may not be converting ADO into inosine, which has immunomodulatory activity (62). However, the functional consequence of this impaired termination was not clinically assessed, nor were the levels of adenosine kinase.

CD73 appears to play a significant role in the pathogenesis of EAE, as CD73 ablation was shown to be protective in EAE induced in CD73 knockout mice as opposed to producing detrimental inflammatory effects, as was initially hypothesized. These animals displayed reduced EAE severity (31, 63) and reduced immune cell infiltration in the brain (31) and spinal cord (63), but displayed CD4^+^ T cells secreting more PICs, which enabled a more severe EAE when transferred to CD73^+/+^ T-cell deficient mice in comparison to the transfer of T cells from WT mice. Moreover, the transfer of CD4^+^CD73^+^ T cells from WT mice to CD73^-/-^ recipient mice allowed for EAE induction, which suggests that CD73 must be expressed in CD4^+^ T cells to allow disease induction to occur. Interestingly, CD73 must be expressed in both CD4^+^ T cells and in plexus choroid cells to fully and strongly induce EAE in mice. When either CD4^+^ T cells or recipient mice lacked CD73, no significant EAE could be observed in recipient mice (63).

T cell suppression beyond Tregs has also been reported to be mediated by astrocytes. Interestingly, astrocytes are capable of inducing expression of CD39 and CD73 in activated and infiltrating T cells in a contact- and TGF-β-dependent manner (64). Consequently, these T cells acquire an “immunosuppressive phenotype”, as evidenced by their ability to inhibit the proliferation of anti-CD3-stimulated T cells *in vitro* (64). Of note, although these aforementioned CD39^+^CD73^+^ T cells upregulated the expression of the transcription factor RORγt, they did not become pathogenic, as the levels of IL-17A remained low (64). In addition, infiltrating T cells induce the expression of CD39 in astrocytes in a fashion that depends on Rai, the Shc family protein adaptor (65), suppressing their activity. Furthermore, immunosuppression of T cells by astrocytes can also be achieved *via* astrocytic CTLA-4 expression in a ADO-dependent manner (65). Altogether, the bidirectional communication between astrocytes and infiltrating encephalitogenic T cells mediated by ectonucleotidases and ADO dysfunction are of paramount importance to the development of MS. Although the activity status of proteins involved in the termination of ADO signaling (ENTs and the cytoplasmic enzymes such as adenosine kinase) is not known in MS, it is reasonable to state that excess ADO signaling through extracellular ADO accumulation drives inflammation and MS progression. In contrast, decreased plasma ADO levels and in PBMCs also allows an excessive inflammatory response to occur, which is detrimental. In this regard, the development of therapies targeting the adenosinergic signaling pathway must consider these different compartments (CSF/CNS parenchyma versus peripheral blood, for instance). Therapies promoting ADO termination or decreasing the activity of CD39 and CD73 may likely be useful in ameliorating MS when used to target the CSF/CNS parenchyma. However, therapies stimulating ADO production may be more useful in the context of peripheral blood and immune cells. Of note, caution should be taken when targeting these enzymes as prevention of the physiological actions of ADO would be damaging. Consequently, unwanted side-effects could occur, which may limit the use of this strategy in the treatment of MS.

Expression of CD39 in microglia/macrophages and brain-infiltrating mononuclear cells may act as a further mechanism for the inhibition of neuroinflammation in MS. Apart from CD39 expression in astrocytes, it was shown that spinal cord microglia/macrophages expressed high levels of CD39 and Arg-1, an M2 marker, at disease peak in comparison to disease onset, during which the M1-phenotype prevailed. When resolution of neuroinflammation in this acute monophasic disease model occurred, CD39 levels returned to basal levels (60).

## Implications for behavioral changes in MS

In addition to neurological symptoms, MS is characterized by multiple neuropsychiatric co-morbidities, such as anxiety and depression (66). Numerous studies have demonstrated that in the asymptomatic phase of EAE, namely before the onset of motor dysfunction, there are extensive behavioral changes, such as anxiety- and depressive-like behavior, which mimics what is observed in MS (66). Of note, the adenosinergic signaling has been implicated in the pathogenesis and treatment of major depressive disorder (MDD) (67). Therefore, it would be expected that some degree of dysfunction in this signaling pathway would occur as a mechanism underpinning these behavioral changes. In monophasic EAE, no changes in the activity of CD39 and CD73 were detected in the serum or spinal cord of rats on dpi 8 (59). However, high levels of CX3CL1 induced by A2AR were detected on dpi 10 (31). It is reasonable to hypothesize that the infiltration of T lymphocytes and macrophages on dpi 10 may drive behavioral changes observed in the absence of motor dysfunction, as these cells have been extensively implicated in the pathophysiology of MDD and other neuropsychiatric disorders (68). Furthermore, augmented levels of A3R were found in the lymph nodes and spleens of mice on dpi 5 (33) and high expression of A2BR was found on dendritic cells on dpi 3-9, but the functional implication of these findings and their relevance to the behavioral dysfunction that precedes the motor dysfunction in MS remains to be elucidated. More studies are required to elucidate the role of the adenosinergic signaling pathway in the behavioral changes observed in MS and EAE.

## Conclusion and future perspectives

**Figure 1:**
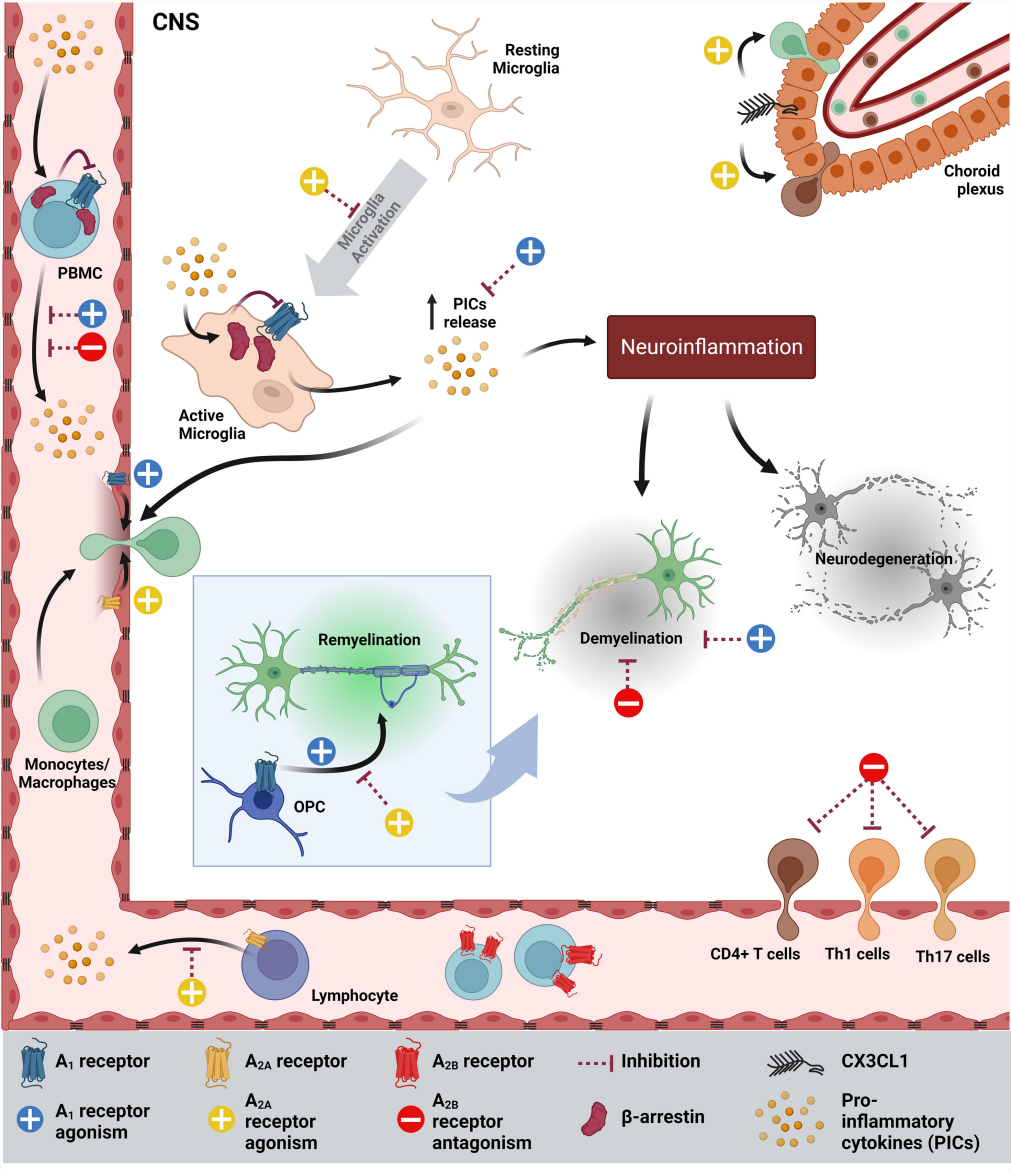
Schematic summarizing the how adenosinergic signaling contributes to MS pathology and how it could be targeted to improve MS symptoms. PICs inhibits the expression of A1R, while increasing the levels of β-arrestin, which further inhibits A1R in microglia and PBMCs. As a result, more PICs are secreted by activated microglia, contributing to neuroinflammation, demyelination and neurodegeneration. Agonism of A1R increases the permeability of the blood-brain barrier (BBB), OPC maturation, migration and myelination and inhibits demyelination. In MS, A2AR is highly expressed on endothelia/lymphocytes and A2AR agonism inhibits microglia, CD4+ T cell migration, increases BBB permeability, inhibits OPC differentiation and increases CX3CL1, which facilitates immune cell infiltration into the CNS. On the other hand, A2AR antagonism decreases CX3CL1 expression. In MS and EAE, A2BR expression in PBMCs/spleen/lymph node (LN) is high (not shown) and A2BR antagonism reduces demyelination, the Th1 and Th17 infiltration into the CNS, PICs and IL-6 in DCs (not shown). CNS, Central Nervous System.

In conclusion, the adenosinergic signaling pathway provides another avenue towards establishing a more integrated understanding of the MS pathology (**Figure 1**) (**Table 1**), which is essential to the development of new therapeutics. It is evident that the correct timing and disease type (RRMS or progressive MS) will dictate the type of therapy (whether agonistic or antagonistic) that will be effective in improving MS symptoms, which are in part caused by a malfunction in this signaling pathway. With the exception of A2BR, targeting all other P1Rs may cause unwanted and paradoxical side effects, such as impairment in myelination, immune cell infiltration and increased BBB permeability. The extent to which these changes may occur needs to be determined in rodent models and in the clinical setting to corroborate whether targeting A1R and A2AR, for instance, is a viable therapeutic option for MS patients.

**Table 1:** Summary of the main features of the adenosine receptors and the effect of its activation or inhibition by agonists and antagonists in the context of MS.

**Receptor type**	**Main features**	**Agonism**	**Antagonism**
**A1R**	Activated by pro-inflammatory cytokines (PICs) and triggers the production of PICs; increased in PBMCs of MS patients; modulates glutamatergic and GABAergic synapses	Increases the permeability of the BBB, the maturation of OPCs and myelination; inhibits demyelination; triggers activation of K+ channels	Not specified
**A2AR**	Regulation of excitatory neurotransmission and synaptic plasticity; Highly expressed in the endothelium and lymphocytes of MS patients; inhibition of OPC differentiation	Inhibition of PICs secretion, of VLA-4 and NFkB; inhibition of phagocytosis and migration of microglia/macrophages; inhibition of migration of CD4+ T lymphocytes; attenuates EAE development (early disease); worsen of EAE (late disease); prevents BBB permeability; increases the BBB permeability; triggers the expression of CX3CL1/Fractalkine; increased the expression of the CCL20 gene.	Inhibition of immune cell infiltration into the CNS, demyelination and PICs secretion (after disease onset); inhibits the expression of CX3CL1/Fractalkine; inhibits immune cell infiltration into the CNS; decreases the expression of CLL20 and Th17 migration into the CNS
**A2BR**	Regulation of excitatory neurotransmission and synaptic plasticity; highly expressed in PBMCs of MS patients and in the spleen and lymph nodes in EAE; highly activated in pathological conditions.	Not specified	Inhibits demyelination and immune cell infiltration into the CNS; Decrease the frequency of Th1 and Th17 cells; inhibits the secretion of PICs and DC derived IL-6;
**A3**	Modulation of immunity; highly expressed in the spinal cord of EAE mice	Inhibition of PICs secretion by microglia	Not specified
**CD39**	Highly expressed in PBMCs and CSF of MS patients (relapse); reduced expression in Tregs from MS patients; highly expressed in the spinal cord of EAE mice	Not specified	Not specified
**CD73**	Highly expressed in PBMCs and CSF of MS patients (relapse); reduced expression in Tregs from MS patients; highly expressed in the spinal cord of EAE mice	Not specified	Not specified
**ADA**	Highly expressed in PBMCs and CSF of MS patients (relapse); reduced expression in Tregs from MS patients; highly expressed in the spinal cord of EAE mice	Not specified	Not specified

Given the paucity of studies on A3R and on the proteins involved in the termination of ADO signaling, future studies should focus on the role of A3R, ENTs, ADA and adenosine kinase to elucidate their role in MS disease pathogenesis. Furthermore, considering the key role of the gut microbiota in the pathogenesis of MS and its direct role in the modulation of the ectonucleotidase CD39, future studies should also focus on the role of the gut microbiota and its metabolites in the adenosinergic signaling pathway, as this would allow, for instance, the modulation of this pathway by pre- and probiotics and other gut microbiota-targeted therapies and the development of new alternatives to treat MS in a gut microbiota and adenosinergic signaling-directed fashion.

## Author contributions

ED-S conceived the study, performed the literature search, data collection, data analysis, and wrote the manuscript under the supervision of CP. HU, ÁO-G, H-PH and SGM critically reviewed and edited the manuscript. ED-S drafted the and ÁO-G created the figure. All authors approved the final version of this manuscript.

## Funding

The authors express their gratitude to Oswaldo Cruz Foundation of Pernambuco (FIOCRUZ-PE), Research Excellence Program - Aggeu Magalhães Institute (IAM PROEP#400208/2019-9), Knowledge Generation Program – Oswaldo Cruz Foundation (FIOCRUZ; #VPPCB-007-FIO-18-2-17), the Brazilian National Institute of Science and Technology on Neuroimmunomodulation (INCT-NIM; #465489/2014-1), the Brazilian National Council for Scientific and Technological Development (CNPq; #301777/2012-8 and 306392/2017-8) and São Paulo Research Foundation (FAPESP; #2018/07366-4 and 2018/08426-0) for research support. ÁO-G is grateful for a postdoctoral fellowship granted by FAPESP (#2019/26852-0). This study was funded in part by the Coordenação de Aperfeiçoamento de Pessoal de Nível Superior - Brasil (CAPES) - Finance Code 001. The funders had no role in study design, data collection and analysis, decision to publish, or preparation of the manuscript.

## Conflict of interest

The authors declare that the research was conducted in the absence of any commercial or financial relationships that could be construed as a potential conflict of interest.

## Publisher’s note

All claims expressed in this article are solely those of the authors and do not necessarily represent those of their affiliated organizations, or those of the publisher, the editors and the reviewers. Any product that may be evaluated in this article, or claim that may be made by its manufacturer, is not guaranteed or endorsed by the publisher.
